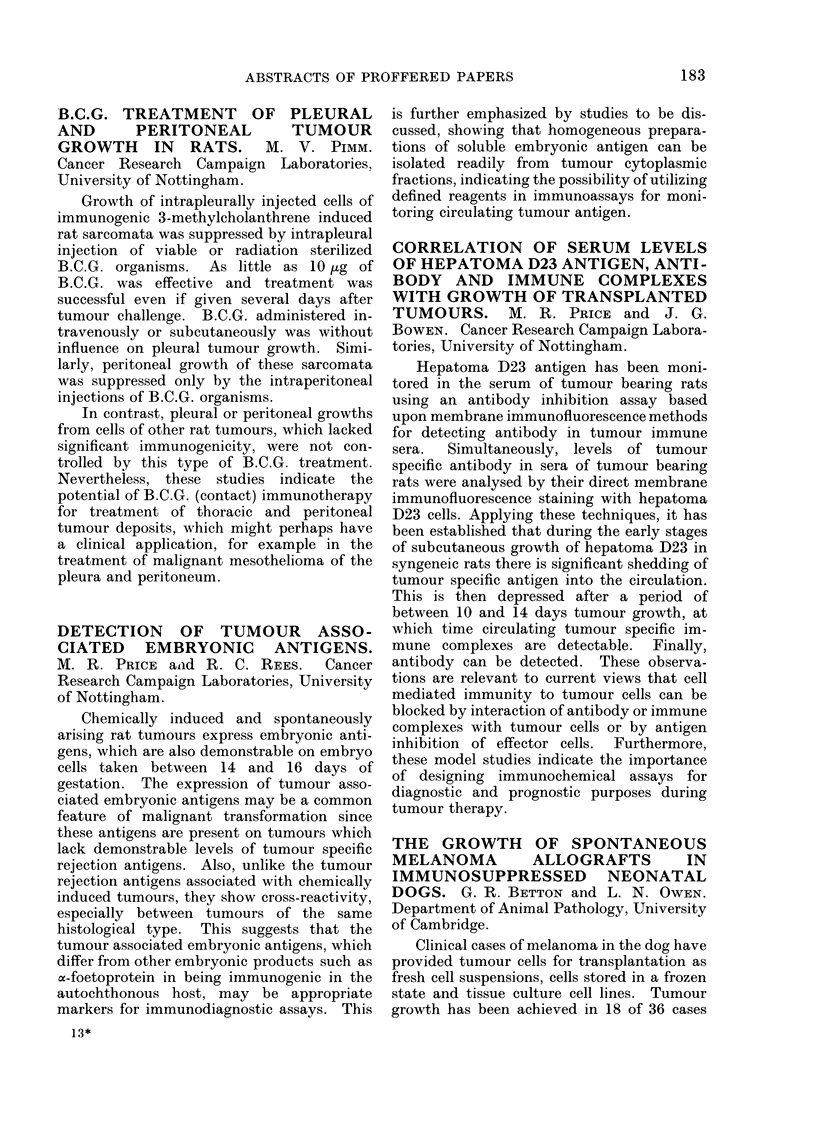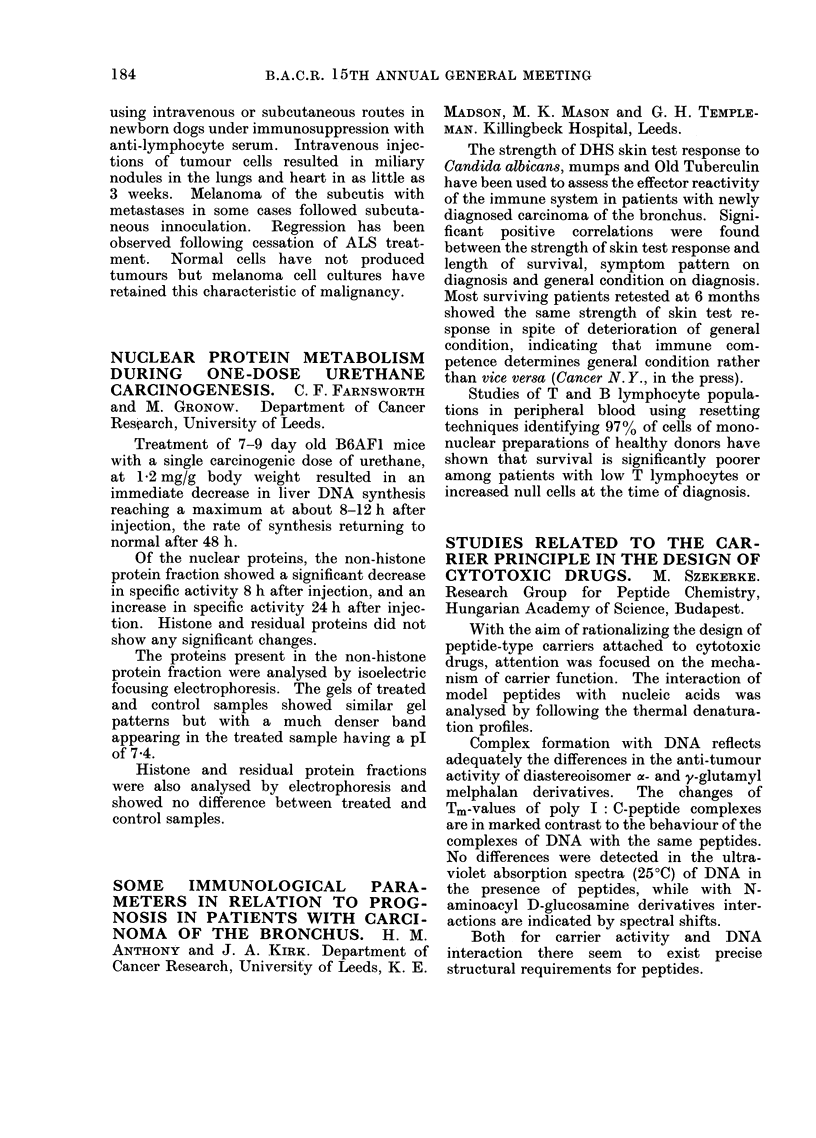# Proceedings: The growth of spontaneous melanoma allografts in immunosuppressed neonatal dogs.

**DOI:** 10.1038/bjc.1974.168

**Published:** 1974-08

**Authors:** G. R. Betton, L. N. Owen


**THE GROWTH OF SPONTANEOUS MELANOMA ALLOGRAFTS IN IMMUNOSUPPRESSED NEONATAL DOGS.** G. R. BETTON and L. N. OWEN. Department of Animal Pathology, University of Cambridge.

Clinical cases of melanoma in the dog have provided tumour cells for transplantation as fresh cell suspensions, cells stored in a frozen state and tissue culture cell lines. Tumour growth has been achieved in 18 of 36 cases using intravenous or subcutaneous routes in newborn dogs under immunosuppression with anti-lymphocyte serum. Intravenous injections of tumour cells resulted in miliary nodules in the lungs and heart in as little as 3 weeks. Melanoma of the subcutis with metastases in some cases followed subcutaneous innoculation. Regression has been observed following cessation of ALS treatment. Normal cells have not produced tumours but melanoma cell cultures have retained this characteristic of malignancy.